# A Systematic Review and Meta-Analysis on Oral Health Disparities Among the Indigenous Paediatric Population

**DOI:** 10.7759/cureus.41673

**Published:** 2023-07-10

**Authors:** Ankita Verma, Harsh Priyank, Renuka P, Minti Kumari, Nishath Sayed Abdul, Sahana Shivakumar

**Affiliations:** 1 Department of Pedodontics and Preventive Dentistry, Hazaribag College of Dental Sciences and Hospital, Ranchi, IND; 2 Department of Conservative, Endodontics and Aesthetic Dentistry, Dental Institute Rajendra Institute of Medical Sciences, Ranchi, IND; 3 Department of Pedodontics and Preventive Dentistry, Government Dental College, Dibrugarh, IND; 4 Department of Public Health Dentistry, Patna Dental College and Hospital, Patna, IND; 5 Department of Oral Pathology, College of Dentistry, Oral Diagnostic Sciences, Riyadh Elm University, Riyadh, SAU; 6 Department of Public Health Dentistry, People's College of Dental Science and Research Center, Bhopal, IND

**Keywords:** dental caries, indigenous, ethnic disparities, oral health disparities, childhood, oral health

## Abstract

There is a knowledge gap in the literature regarding oral health disparities (OHD) in minority and indigenous (IG) paediatric cohorts that needs to be addressed. Disparities in oral health among children are a pressing concern, highlighting inequities in access to dental care and meeting needs. The current systematic review aims to provide a comprehensive synthesis of the prevailing understanding of OHD in the minority and IG strata.

A meticulous search strategy was formulated by a team of reviewers to identify pertinent studies from databases of PubMed, MEDLINE, Scopus, Google Scholar and EMBASE. Data extraction and article selection strictly adhered to Preferred Reporting Items for Systematic Reviews and Meta-Analyses (PRISMA) guidelines. The Newcastle-Ottawa Scale (NOS) was employed to evaluate the methodological quality of the studies included. Review Manager version 5.4 was used to synthesise quantitative data. A total of five cross-sectional studies were included in the final analysis. The findings consistently demonstrated the existence of racial and socioeconomic disparities in oral health across varying age groups and geographical locations in the defined population. Significant disparities in oral health outcomes were observed between IG and non-IG populations, with IG and minority groups exhibiting a heightened vulnerability to oral health challenges. Through a meta-analysis of the compiled data, a statistically significant association was established between children (being a member of a minority group) and unmet oral health needs. Socioeconomic status (SES) and maternal education were factors that showed a significant impact on oral health disparity. All studies were graded to be of the low-risk category based on the NOS risk of bias tool.

This review successfully identified several influential factors contributing to oral health disparities, such as cultural practices, dietary patterns and access to oral healthcare services. Additionally, discernible differences in oral health status were evident between IG and non-IG children, with IG children enduring a greater burden of oral health difficulties. These findings underscore the imperative for targeted interventions and policy measures aimed at addressing the specific oral health needs of minority and IG paediatric populations, with the overarching goal of mitigating the existing disparities.

## Introduction and background

In many countries, indigenous (IG) groups are a minority population, and they can face significant social, economic and political challenges [1]. They are often subjected to displacement, discrimination and marginalisation by non-IG groups [2]. For example, in Australia, IG Australians make up only around 3% of the total population but experience higher rates of poverty, unemployment and incarceration than non-IG Australians [3]. Similarly, in Canada, IG groups make up around 4% of the population but experience it disproportionately [4].

IG and non-IG groups and minorities living in different countries face a range of health problems, often due to a myriad of factors [5]. For IG communities, these health problems can also be attributed to historical trauma, the loss of traditional lands and cultural practices and forced assimilation. The health disparities faced by these populations are significant and have been well documented [6]. IG populations, for example, have higher rates of infectious diseases [6-8], as well as chronic diseases [9,10]. In Australia, IG Australians have a life expectancy that is approximately 10 years less than that of non-IG individuals [3]. In Canada, IG communities have higher rates of infant mortality, suicide and substance abuse compared to non-IG Canadians [4]. In the United States, IG populations have higher rates of systemic disorders and substance abuse compared to non-native populations [11,12].

Oral health disparities (OHD) have been extensively documented among IG and minority populations when compared to the majority population. Research studies consistently reveal that IG populations experience higher rates of dental caries, periodontal diseases and tooth loss [13-15]. Similarly, minority populations such as African Americans, Hispanics and Asians also exhibit disparities in oral health outcomes. For instance, African Americans and Hispanics have a higher prevalence of untreated dental caries and tooth loss, while Asian Americans demonstrate an elevated incidence of periodontitis [15].

Dental caries is the most prevalent oral disease affecting a significant proportion of school-aged children and nearly all adults [16]. Furthermore, minority populations face challenges in accessing appropriate oral healthcare, resulting in persistent untreated dental caries, periodontal diseases and tooth loss [17]. This disparity is evident among African Americans and Hispanics, who demonstrate lower utilisation of dental care services compared to non-Hispanic whites in the United States [18,19]. The influence of socioeconomic status (SES) on oral health outcomes is well documented [20]. Individuals from lower socioeconomic backgrounds exhibit a higher vulnerability to oral health issues, including dental caries, periodontal diseases and tooth loss [21].

Additionally, research has demonstrated that the oral health issues that IG and minority communities deal with can significantly affect their general health and quality of life [17]. Untreated dental caries and gum conditions, for instance, can result in pain, infection and even tooth loss, which can impair one's capacity for eating, speaking and maintaining a healthy diet [14]. These issues can harm a child's social connections and sense of self-worth, which can result in psychological anguish and social isolation [13].

While there has been a significant amount of research conducted on OHD among children, there are still several gaps in the literature such as limited representation of indigenous paediatric populations and insufficient longitudinal studies [17-22]. One of the main gaps is the lack of research focused specifically on the OHD faced by children from minority and IG populations. While there is some literature available on this topic, there is a need for a more comprehensive review that synthesises the existing evidence. Hence, through this systematic review, we aimed to determine disparities between IG and non-IG child populations and evaluate the factors contributing to oral health disparities, including social determinants of health, cultural factors and socioeconomic status. By addressing these literature gaps and synthesising the available evidence, we hope to contribute to the development of effective interventions and policies aimed at reducing these disparities and improving oral health outcomes for all children. Hence, the current review was undertaken to answer the research question "Is there a disparity in oral health between IG and non-IG paediatric population?"

## Review

Methods

Review Guidelines and Population, Exposure, Comparison and Outcome (PECO)

**Figure 1:**
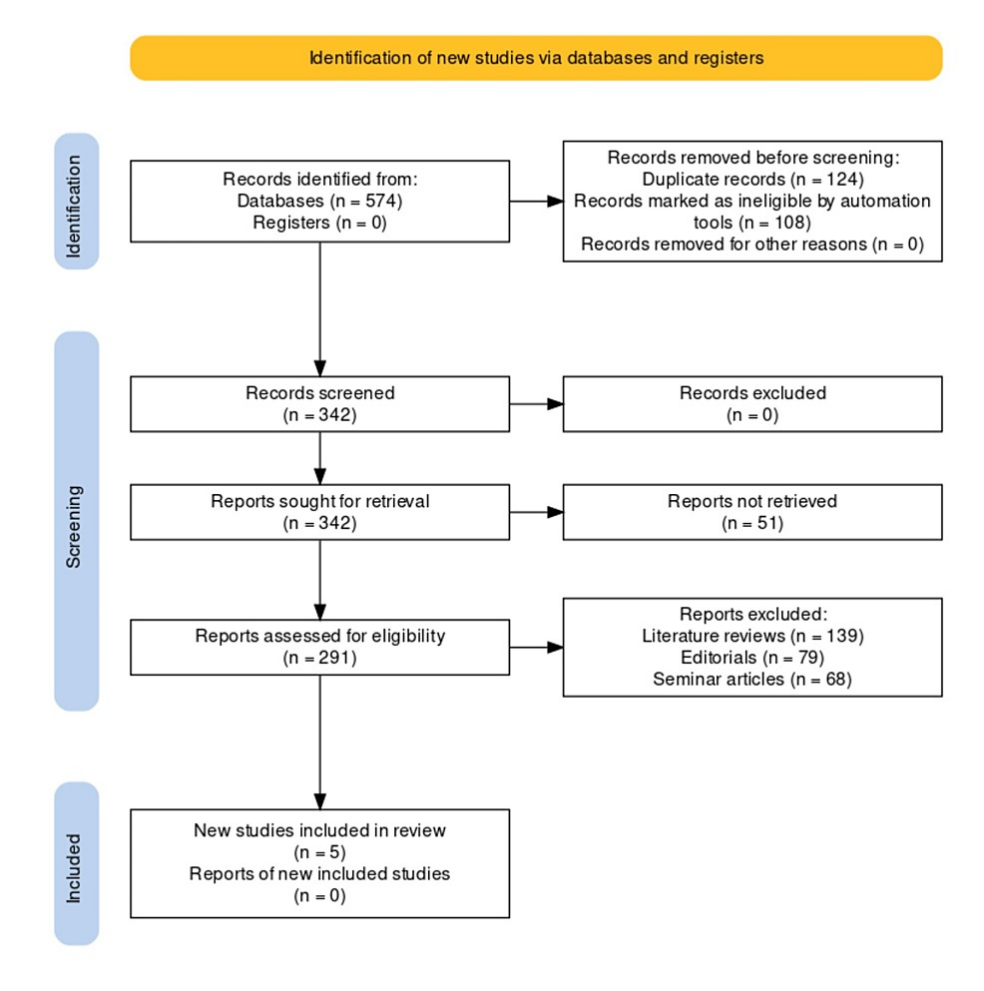
Framework for article selection using PRISMA guidelines PRISMA: Preferred Reporting Items for Systematic Reviews and Meta-Analyses

The review adhered to Preferred Reporting Items for Systematic Reviews and Meta-Analyses (PRISMA) guidelines to ensure the completeness and transparency of reporting, with Figure 1 representing a flow diagram that illustrates the study selection process [23,24]. By utilising these guidelines, we ensured that the review was comprehensive and transparent, increasing the reliability and validity of the study findings.

The Population, Exposure, Comparison and Outcome (PECO) strategy was used to formulate the research question and guide the selection of relevant studies. The population (P) was children of indigenous and minority groups of different ages and ethnicities, exposure (E) was any programme or intervention aimed at improving oral health outcomes, comparison (C) in this review is between the majority and non-indigenous populations and outcomes (O) are effects on oral health, such as decreased dental caries incidence or improved oral hygiene practices. This strategy was used to systematically search and select relevant studies for inclusion in this investigation and guide the analysis and interpretation of the findings.

Search Strategy

A thorough search was conducted across five different databases to generate relevant papers for this review. The search strategy included a combination of Boolean operators and Medical Subject Headings (MeSH) keywords, as shown in Table 1. The results of each database search were compiled and screened for eligibility criteria.

**Table 1:** Strategy for keyword search across different databases

Database	Search keywords
PubMed	("oral health" OR "dental health" OR "tooth health") AND ("child" OR "adolescent") AND ("health status disparities" OR "socioeconomic factors")
MEDLINE	("oral health" OR "dental health" OR "tooth health") AND ("child" OR "adolescent") AND ("health status disparities" OR "socioeconomic factors")
Scopus	TITLE-ABS-KEY ("oral health" OR "dental health" OR "tooth health") AND TITLE-ABS-KEY ("child" OR "adolescent") AND TITLE-ABS-KEY ("health status disparities" OR "socioeconomic factors")
Google Scholar	("oral health" allintitle:"dental health" OR allintitle:"tooth health") AND ("children" OR "pediatric" OR "childhood") AND ("health status disparities" OR "socioeconomic factors")
EMBASE	"((oral health) OR (dental health) OR (dental care)).mp. AND ((child) OR (children) OR (pediatric)).mp. AND ((disparities) OR (inequalities) OR (disadvantaged)).mp. AND ((systematic review) OR (meta-analysis) OR (review))

Eligibility Criteria

Studies published in peer-reviewed journals, written in English, conducted on indigenous populations up to 18 years old and reporting on OHD among children were assessed further in this review. Various study designs, including cross-sectional, cohort, case-control and RCTs, were considered for inclusion. Studies that reported on factors associated with OHD such as SES, race, ethnicity and geographic location were also eligible for inclusion. The exclusion criteria for this study were studies conducted on adults, animals or non-human subjects and studies published in languages other than English. Studies with insufficient data or low quality that did not meet the inclusion criteria were also excluded. Additionally, studies with incomplete information, duplicate studies or studies with inconsistent or unclear reporting were also excluded.

Data Extraction and Risk of Bias Assessment

Two reviewers screened the studies, employing pre-established inclusion and exclusion criteria. In instances of discordance between the two reviewers, a third reviewer was consulted. Reviewers independently extracted data utilising a predefined data extraction instrument. This instrument facilitated the collection of diverse data elements, including study characteristics, study design, participant attributes, details regarding interventions or exposures, outcome measures and findings. Any disparities encountered during the data extraction process were resolved through thorough deliberation between the reviewers. Ultimately, the amassed data underwent synthesis and statistical analysis, employing appropriate methodologies to ascertain the overall effect size and statistical significance of the obtained findings.

The Newcastle-Ottawa Scale (NOS) [25,26] was used to evaluate the risk of bias in this review. The NOS evaluates studies based on three key domains: selection of study groups, comparability of groups and ascertainment of outcomes or exposure. Within these domains, specific criteria are used to assign scores to different components of the study, such as representativeness of the exposed cohort, comparability of study groups and assessment of outcomes. Based on a defined set of standards, each domain was assessed. This instrument has been demonstrated to be a viable and reliable tool for evaluating the calibre of non-randomised investigations.

Statistical Analysis

Forest plots were generated using Review Manager version 5.4 to visualise the results of the meta-analysis. The fixed-effect model was used in the present review with the assumption that the true effect size is the same across all studies being analysed. It assumes that any observed variation in effect sizes between studies is due solely to random error or sampling variability. The forest plots displayed the odds ratio (OR) and 95% confidence interval (CI) for various aspects of OHD among children, as well as the combined OR and its corresponding 95% CI. The overall effect size was assessed for statistical significance by reporting the p-value. The 95% CI for the ORs was computed using the Mantel-Haenszel method. A significance level of alpha = 0.05 was set, indicating a willingness to accept a 5% risk of erroneously concluding a statistically significant difference between groups in the absence of a true difference.

Results

Study Characteristics

Table 2 provides the demographic summary of five studies [27-31] examining OHD among children from diverse age groups and ethnic backgrounds. The studies aimed to investigate various aspects of oral health among children and the potential disparities that exist. The studies were conducted in Australia, Northern Australia, Britain, Canada and China. All studies employed a cross-sectional design to examine OHD at specific time points. The sample sizes varied across the studies, providing a diverse representation of children from different populations and regions.

**Table 2:** Demographic characteristics of the included papers SES: socioeconomic status, IG: indigenous

Author ID	Year	Country	Sample size	Was SES assessed?	Groups	Age range (in years)	Gender ratio	Design
Haag et al. [27]	2021	Australia	13,544	Yes	IG group (n=485) and non-IG group (n=13,059)	5-10	51.1% males	Cross-sectional
Jamieson et al. [28]	2006	Northern Australia	12,584	Yes	IG group (n=4,414) and non-IG group (n=8,170)	4-13	Unspecified	Cross-sectional
Rouxel et al. [29]	2018	Britain	8,541	Yes	5-year-old group (n=2,217), 8-year-old group (n=2,083), 12-year-old group (n=2,183), 15-year-old group (n=2,058)	5, 8, 12 and 15, respectively	50% males	Cross-sectional
Shi et al. [30]	2018	Canada	5,600	Yes	White group (n=2,944), South Asian group (n=771), Filipino group (n=345), Chinese group (n=301), Black group (n=241), Arab group (n=193), Latin American group (n=166), IG group (n=95) and mixed ethnicity group (n=544)	5-8	48.55% males	Cross-sectional
Yun et al. [31]	2021	China	1,926	Yes	Year 2005 group (n=388) and year 2015 group (n=1,926)	12-year-olds only	49.7% males	Cross-sectional

Main Findings

The findings revealed notable differences in oral health outcomes based on factors such as IG status, SES, age and ethnicity. The studies demonstrated variations in oral health profiles between IG and non-IG children, highlighting the presence of disparities. Additionally, the investigations explored the influence of SES on oral health outcomes among children and identified associations between different ethnic groups and oral health disparities. Three out of five studies attributed unmet oral health needs to SES [27-29]. Maternal literacy was a direct factor in influencing oral health disparity [31].

Table 3 presents a summary of the technical factors related to OHD assessed in this review. Haag et al. [27] studied the Decayed, Missing and Filled Teeth (DMFT) scores of IG and non-IG children aged 5-10 years and found that both groups faced greater disease rates and required more clinical intervention as a result of socioeconomic disadvantage. Jamieson et al. [28] found that ethnicity and socioeconomic status had significant connections with oral health outcomes, with the greatest difference between IG and non-IG children observed among the most disadvantaged groups across all age categories. Rouxel et al. [29] observed that lower socioeconomic status in the family was linked to greater rates of dental decay in younger children, but not in 15-year-olds, and that significant disparities in oral health caused by residential deprivation persisted among adolescents. Shi et al. [30] found that compared to White populations, Filipinos, Arabs and IG communities were more likely to have poor oral health, even after accounting for demographic or socioeconomic factors. Yun et al. [31] noted that between 2005 and 2015, disparities in children's dental visits decreased, but disparities in dental visits and untreated caries related to maternal education were still present.

**Table 3:** Characteristics of the included articles pertaining to the ethnicity of the included sample size, the assessment period and the oral health assessment drawn from the respective papers DMFT: Decayed, Missing and Filled Teeth, IG: indigenous, dmfs: decayed, missing and filled surfaces, SES: socioeconomic status, OHD: oral health disparities

Author ID	Oral health variables assessed	Ethnic demographics assessed	Ethnic relationship with oral health findings	Assessment period	Inference assessed
Haag et al. [27]	DMFT scores	IG and non-IG	dmfs for IG children aged 5-10 was 6.4 (mean), ranging from 9.1 for the lowest income group to 2.3 for the wealthiest. Non-IG children in the lowest economic category had a mean dmfs of 2.9, ranging from 1.9 to 4.2.	2 years	Due to SES inequality, both IG and non-IG groups experienced higher illness rates and needed more clinical intervention, with the social differences being noticeably more prominent in IG children.
Jamieson et al. [28]	Filled and decayed teeth, dental caries (with or without multiple tooth involvement)	IG and non-IG	Five-year-old IG children had mean DMFT levels that were 3.0 times higher than those of non-IG children.	1 year	The findings suggested that racial or ethnic origin affected oral health outcomes independently of SES, suggesting that SES and oral health outcomes had substantial links but were not connected with one another.
Rouxel et al. [29]	Filled and decayed teeth, plaque levels, gingival health and periodontal status	Indian, Pakistani, Bangladeshi, White British and Irish, Black Caribbean, Black African and mixed White	By the age of 15, ethnic differences had significantly decreased. Greater rates of tooth decay were associated with lower household socioeconomic position in younger children, but not in 15-year-olds. Additionally, compared to the lower ages, the 15-year age group had larger periodontal disparities.	1 year	British teens displayed less variance in oral health according to race and familial SES than younger children did. Significant OHD caused by residential disadvantage did, however, continue among teenagers.
Shi et al. [30]	Parental observations of below optimal/suboptimal oral health, filled and decayed teeth and dental caries	Caucasian, Indo-Aryan, Tagalog, Han Chinese, African, Arab, Latin American, IG and multiracial	Compared to White children, Filipino children had about fivefold greater odds of having serious untreated dental issues, especially with respect to dental caries in multiple teeth.	1 year	Compared to White populations, Filipinos, Arabs and IG communities were more likely to have worse levels of oral health.
Yun et al. [31]	Untreated caries	The majority and minority population (comparison was also based on differences in income-based and maternal education status)	The prevalence of untreated caries reduced in the assessed time period, although the number of dental visits among 12-year-old kids increased.	10 years	OHD reduced during the evaluated time period for paediatric dental visits. OHD connected to maternal education was still evident, though.

Metanalytic Results

**Figure 2:**
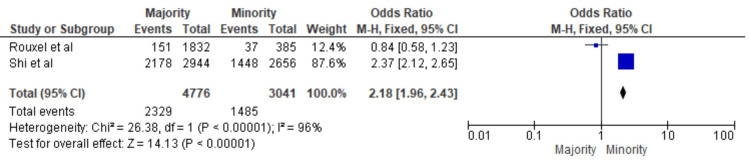
Overall impact of DMFT scores of the majority versus minority groups of children on oral health in the selected studies* *[29,30] DMFT: Decayed, Missing and Filled Teeth, CI: confidence interval

Figure 2 represents the overall impact of DMFT scores for the majority versus minority groups of children on oral health in the selected studies. The odds of IG children having poorer oral health than non-IG children are 2.18 times higher (95% CI: 1.70-2.04).

**Figure 3:**
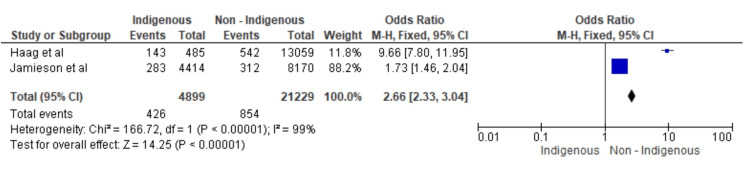
Overall impact of DMFT scores of IG versus non-IG groups of children on oral health in the selected studies* *[27,28] DMFT: Decayed, Missing and Filled Teeth, IG: indigenous, CI: confidence interval

Figure 3 represents the overall impact of DMFT scores on IG versus non-IG groups of children. An OR of 2.18 with a 95% CI of 1.96-2.43 was noted, which indicates that there is a significantly higher likelihood of children belonging to the IG group having poor oral health as compared to the majority group. The heterogeneity test statistics show that there is a significant amount of heterogeneity in the selected studies, which could be attributed to methodological differences, reporting variance and geographical variation. Overall, the results suggest that belonging to a minority group is associated with a higher risk of poor oral health in children.

Quality Assessment of the Studies Included

**Figure 4:**
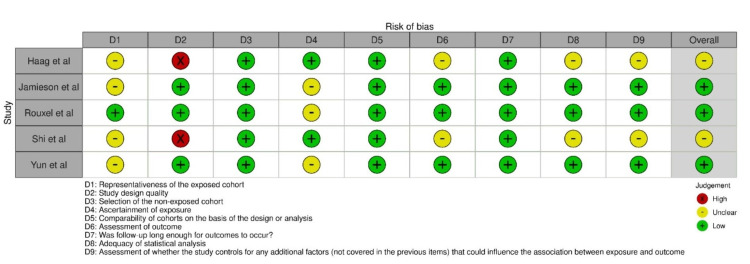
Assessment of risk of bias in the studies selected for this review* *[27-30]

All the studies were graded as moderate risk, with scores ranging from 5 to 9. The studies of Haag et al. [27] and Shi et al. [30] showed some concerns in the final assessment, while the other three were of low risk, as seen in Figure 4.

Discussion

The significance of this review lies in its contribution to the understanding of OHD among children of different ages and ethnic groups. The review compiled and analysed data from five different studies conducted across various countries, providing a broad and comprehensive overview of the issue. The findings of the study suggest that ethnicity, socioeconomic status and other demographic factors play a crucial role in determining oral health status among children. The study also highlights the complex interplay between these factors, indicating that targeted interventions are necessary to address the disparities. The clinical implications of this study are significant. With this knowledge, they can design and implement targeted interventions to improve oral health outcomes in disadvantaged communities. Additionally, the study's findings can help practitioners develop strategies to better engage and educate families about oral healthcare practices. The relevance of this study extends beyond the dental community. The study's findings can also inform public health policy and practice, as poor oral health is associated with a range of health problems, including cardiovascular disease, diabetes and respiratory illness. By addressing OHD among children, public health practitioners can improve overall health outcomes and reduce the burden of disease on communities. All in all, this study provides valuable insights into the factors that contribute to OHD among children. The study's findings have important clinical and public health implications, highlighting the need for targeted interventions to improve oral health outcomes in disadvantaged communities.

Empirical evidence reveals that children and adolescents from lower SES backgrounds are at a heightened risk of experiencing suboptimal oral health outcomes compared to their counterparts from higher SES backgrounds. Various factors contribute to this disparity. Limited access to preventive oral healthcare services, such as regular dental check-ups and fluoride treatments, is prevalent among children from low-income families [20,21]. Consequently, their vulnerability to dental caries and other oral health issues is exacerbated. Furthermore, their dietary habits often involve a high intake of sugar and a lack of essential nutrients, further increasing the likelihood of tooth decay [32]. Additionally, adolescents from lower SES backgrounds are more likely to engage in behaviours detrimental to oral health, such as smoking and consuming sugary beverages [33]. Their oral health knowledge levels may also be deficient, impacting their oral hygiene practices [33].

In terms of ethnic and socioeconomic differentials in oral health, investigations have explored these associations across various age cohorts. Notably, cross-sectional evidence suggests that among Danish adolescents, ethnic differences in oral health appear to be relatively smaller [34]. Similarly, studies conducted among US [35] and French adolescents [36] indicate a reduction in socioeconomic disparities compared to younger children. However, it is important to note that previous studies have not explicitly examined the hypothesis of socioeconomic equalisation in oral health during adolescence. Furthermore, longitudinal data from New Zealand reveals that the significant disparities observed in primary dentition at age 5 exhibit a slight reduction by age 18, followed by a re-emergence and widening of disparities by age 26. Late childhood and early adolescence represent critical developmental stages characterized by transitioning from a family-centred environment to a broader social milieu influenced by peers and external factors [37].

The health outcomes of adolescents are intricately shaped by the scholastic milieu, acting as both an equalizing force in mitigating health disparities and an agent that perpetuates new disparities with long-standing implications [38]. Our recent investigation elucidated a compelling association between adolescents hailing from economically disadvantaged residential areas and compromised oral health, whereas the prevalence of dental caries among younger children exhibited a robust correlation with family-based SES [39]. Moreover, a salient observation emerged linking children residing in more deprived localities to proximity to fast-food establishments, a circumstance that concomitantly corresponded to heightened rates of overweight and obesity among 10-11-year-olds. The burgeoning autonomy of these older children in determining their dietary choices played a pivotal role in this phenomenon [40,41].

The transitional phase from childhood to adolescence ushers in transformative shifts in oral health behaviours, whereby parental oversight of tooth brushing recedes while peer groups and media influence ascend in prominence [42]. Furthermore, the expanded accessibility to sugar-laden foods and beverages exerts a tangible impact on the oral health status of adolescents [43]. Intriguingly, our inquiry unmasked nuanced alterations in oral health behaviours across distinct ethnic and socioeconomic groups during this pivotal life stage [44]. White British adolescents exhibited a predilection for engaging in precarious health behaviours when juxtaposed with their ethnic minority counterparts [45]. Furthermore, a notable socioeconomic chasm in toothbrushing practices emerged, progressively widening as adolescents traverse the developmental trajectory from 12 to 15 years of age. However, the trajectory of water consumption revealed a divergent pattern, wherein disadvantaged 12-year-olds displayed higher levels of water intake in comparison to their advantaged 15-year-old peers [46].

Limitations

Several limitations persist despite the informative insights offered by the chosen studies. As the studies evaluated were all of cross-sectional design, a causal connection between the outcomes of oral health and demographic factors cannot be established. Direct comparison of the results is challenging because the research's sample sizes, age ranges and definitions of demographic characteristics vary. Furthermore, measurement bias might have been introduced because the studies utilised various evaluation techniques to evaluate oral health outcomes. For instance, whereas some research focused on clinical data, others used self-reported data, which could result in either an under- or over-reporting of oral health issues. Moreover, the association between demographic characteristics and oral health outcomes may be impacted by potential confounders such as dietary practices, mouth hygiene routines or access to oral healthcare, which were not taken into account in some studies.

## Conclusions

Our review sheds light on the OHD among children belonging to different ethnicities and socioeconomic backgrounds. We identified several key factors contributing to these disparities, including access to oral healthcare, dietary habits and cultural practices. Our review also revealed notable fluctuations in the oral health status of IG and non-IG children, with IG children facing greater oral health challenges. Furthermore, our review highlighted significant literature gaps in this area, particularly with respect to the effectiveness of various interventions aimed at reducing OHD among children. We aimed to address these gaps by providing a comprehensive analysis of the existing literature and generating evidence-based recommendations for future research and policy interventions.
